# Extensive Upper Airway Hematoma Secondary to Supratherapeutic Warfarin Anticoagulation

**DOI:** 10.5811/cpcem.2020.7.48695

**Published:** 2020-10-19

**Authors:** Taofiq Olusegun Oyedokun, Kevin Manuel Durr

**Affiliations:** * University of Saskatchewan, Department of Emergency Medicine, Saskatoon, Saskatchewan, Canada; † University of Ottawa, Department of Emergency Medicine, Ottawa, Ontario, Canada

**Keywords:** anticoagulation, swelling, airway, hematoma

## Abstract

**Case Presentation:**

A 63-year-old female presented to the emergency department complaining of cough, neck swelling, dysphagia, and dysphonia for two days, with a past medical history of atrial fibrillation managed with warfarin. Investigations revealed a supratherapeutic international normalised ratio (greater than 10). Imaging and endoscopic examination showed an extensive retropharyngeal hematoma with significant mass effect on the airway.

**Discussion:**

A rare but potentially fatal complication of warfarin anticoagulation is upper airway hematoma, with violent coughing described as an inciting cause. Signs of airway compromise necessitate specialist consultation and definitive airway management, while mild cases without airway concerns can be managed conservatively with medical anticoagulation reversal.

## CASE PRESENTATION

**Image 1:**
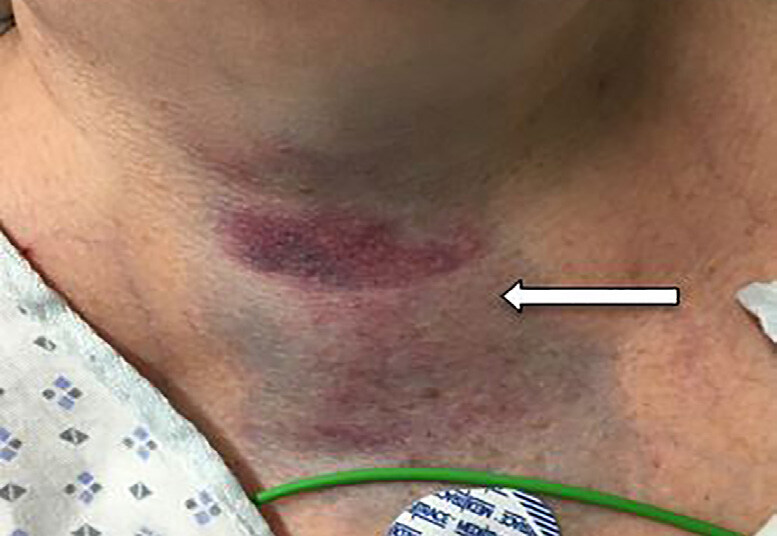
General inspection revealing ecchymosis over the anterior aspect of the patient’s neck (arrow).

**Image 2:**
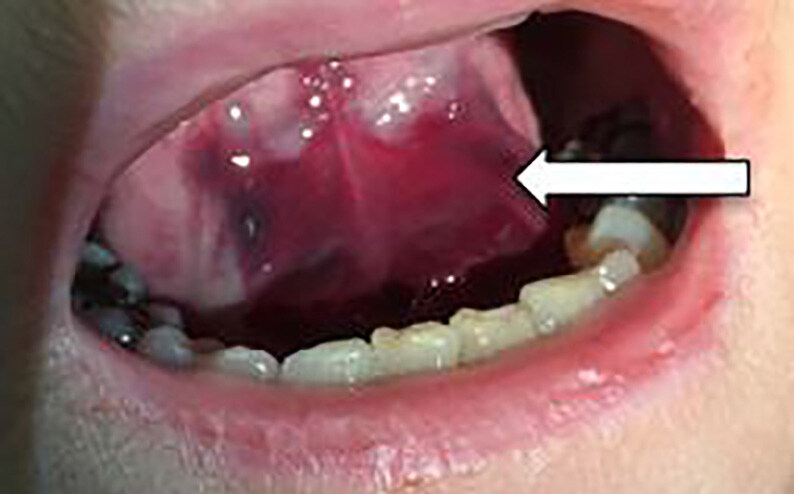
Airway assessment revealing a sublingual hematoma (arrow).

A 63-year-old female presented to the emergency department with a two-day history of cough, neck swelling, dysphagia, and dysphonia. She was taking warfarin for atrial fibrillation. Vital signs were normal, and she was not in respiratory distress. Further examination revealed hoarseness and multiple ecchymoses over the anterior aspect of her neck (Image 1), as well as a sublingual haematoma (Image 2).

**Image 3:**
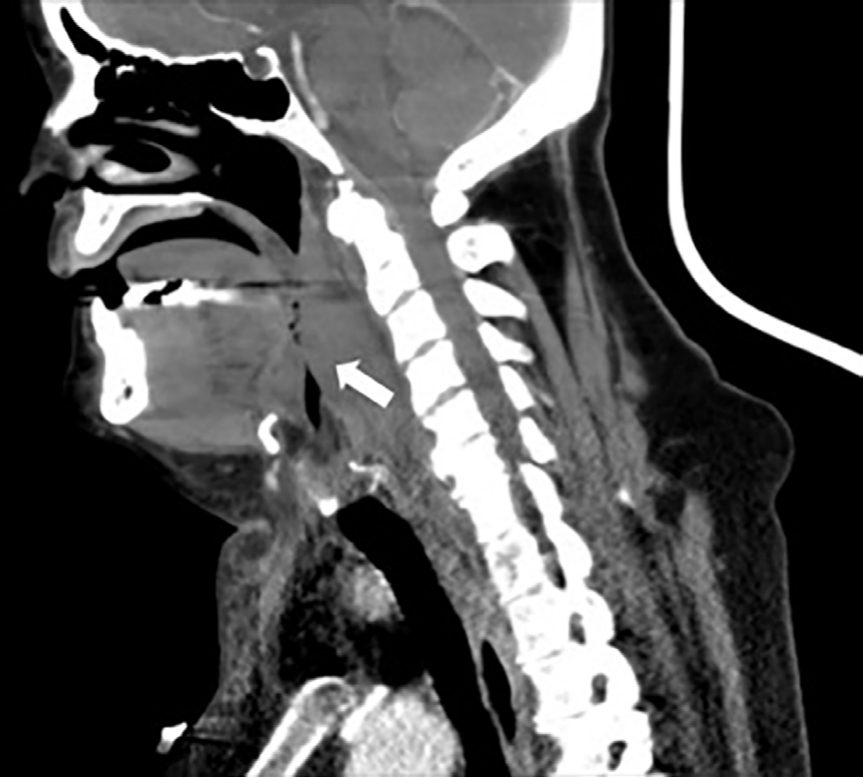
Sagittal view of a non-contrast computed tomography of the neck demonstrating an extensive retropharyngeal hematoma causing mass effect on the airway (arrow).

Immediate anesthesiology and otorhinolaryngology consultations were requested. Non-contrast computed tomography and endoscopic examination revealed a hematoma extending from the retropharyngeal space of the suprahyoid neck, down into the infrahyoid neck to the level of the thyroid cartilage, causing significant mass effect on the airway and right vocal cord (Image 3).

The patient’s international normalised ratio (INR) was supratherapeutic, measuring greater than 10. She was managed conservatively, with 10 mg of vitamin K, 3000 units of prothrombin complex concentrate (PCC), as well as 10 milligrams of dexamethasone, and observed in the intensive care unit (ICU). She was transferred to the surgical observation unit the following day, and discharged home six days later.

## DISCUSSION

Upper airway hematomas are a potentially fatal albeit rare complication of warfarin anticoagulation, with violent coughing described as an inciting cause.^1^ Dysphagia, sore throat, neck swelling, ecchymosis, and hoarseness are common presenting symptoms.^1^ Optimal management remains debated. Signs of airway compromise necessitate timely consultation of anesthesiology and otorhinolaryngology and may require definitive airway management, while mild cases without airway concerns can successfully be managed with medical therapy.^1^,^2^ A combination of vitamin K and fresh frozen plasma (FFP) or PCC, with observation in the ICU is recommended.^1^,^3^ In the context of warfarin-induced coagulopathy, PCC is associated with faster reversal, as well as fewer red cell transfusions and adverse events, relative to FFP.^4^ The recommended PCC dose is four units per kilogram (U/kg) with an INR greater than 1.5 and 50 U/kg with an INR greater than six.^1^

CPC-EM Capsule

What do we already know about this clinical entity?

*Upper airway hematoma is a rare but potentially fatal complication of warfarin therapy*.

*Signs of airway compromise necessitate prompt airway management*.

What is the major impact of the image(s)?

*Initial clinical appearance of unexplained ecchymoses in anticoagulated patients should increase the suspicion of a hematoma in the airway*.

How might this improve emergency medicine practice?

*Rapid reversal of anticoagulation with vitamin K and prothrombin complex concentrate may prevent the need for airway intervention*.
